# Does Body Checking Regulate Emotions? An Experimental Study on Appearance- and Health-Related Body Checking

**DOI:** 10.32872/cpe.15691

**Published:** 2026-02-27

**Authors:** Vanessa Hofschröer, Maj-Britt Vivell, Andrea S. Hartmann, Silja Vocks

**Affiliations:** 1 Department of Psychology, Clinical Psychology and Psychotherapy, Osnabrück University, Osnabrück, Germany; 2 Department of Psychology, Clinical Psychology and Psychotherapy of Childhood and Adolescence, University of Konstanz, Konstanz, Germany; Friedrich-Alexander-Universität Erlangen-Nürnberg, Erlangen, Germany; University of Lausanne, Lausanne, Switzerland

**Keywords:** body checking, safety behavior, eating disorder, body dysmorphic disorder, illness anxiety

## Abstract

**Background:**

Body checking (BC) is widespread among healthy populations and in individuals with eating disorders (EDs), body dysmorphic disorder (BDD), and illness anxiety disorder (IAD). Etiological models of these three disorders originate from research on obsessive-compulsive disorder and propose a short-term reduction of negative affect after BC. However, as empirical evidence shows a heterogenous pattern regarding the reduction of negative affect, the primary objective of this study was to test the etiological models in a cross-over laboratory experiment.

**Method:**

After induction of negative affect, *N =* 102 healthy females underwent a 10-min BC task, in which they were randomly assigned to perform ED-, BDD-, or IAD-related BC, and a 10-min control checking condition of checking the characteristics of two vases. Before and after each task, participants completed state questionnaires on affect and disorder-specific pathology.

**Results:**

The results revealed increased negative affect and disorder-specific pathology from before to after BC, but a reduction of these variables after the control checking condition.

**Conclusion:**

Thus, contrary to expectation, the theory explaining reduced negative affect in compulsive checking may not directly be applicable to ED-, BDD-, and IAD-related BC in healthy populations, thus providing evidence of the dysfunctionality of BC in the short term.

## Highlights

- Cognitive-behavioral models propose a short-term reduction of negative affect after body checking.
- Empirical evidence challenges those assumptions for appearance- and health-related body checking.
- In our study, negative affect increased post-checking, highlighting its short-term adverse effect.
- The study highlights the overall dysfunctionality of body checking behaviors.
- Findings suggest current theories on body checking reinforcement mechanisms may require reevaluation.

When faced with a bodily change (e.g., weight gain, skin irregularities, signs of illness), many people inspect their body in order to prevent, avoid, or escape an unpleasant outcome (Helbig-Lang & Petermann, 2010). Although this behavior, termed *body checking* (BC), is widespread and common among nonclinical populations (cf. Baptista et al., 2021; Wilver et al., 2020), the critical, repeated evaluation of one’s body is also a transdiagnostic feature among individuals with various mental disorders such as eating disorders (EDs; e.g., Shafran et al., 2007), body dysmorphic disorder (BDD; e.g., Veale & Riley, 2001), or illness anxiety disorder (IAD; e.g., Abramowitz & Moore, 2007).

Shafran et al. (2004) reported that 92% of women with EDs engage in BC to monitor their body regarding weight (gain) and shape. In ED-related BC, the focus of attention typically lies on weight and the shape of the individual’s most disliked body parts, as well as generally broader body areas, primarily the abdomen and thighs (Shafran et al., 2004). Furthermore, about 80% of women diagnosed with BDD regularly check their bodies (e.g., Buhlmann & Winter, 2011). In contrast to weight and shape, which are focal areas of ED-related BC (Shafran et al., 2004), BC in BDD most often focuses on specific body parts that are self-characterized as flawed (Buhlmann & Winter, 2011), such as facial skin or the nose (Phillips et al., 2005). Unlike appearance-related BC (e.g., as in EDs and BDD), BC in IAD appears to be health-related. So far, no study has analyzed precisely what proportion of patients with IAD perform BC, but it is assumed that persons with IAD frequently check their bodies for signs of illness. In contrast to ED- and BDD-related BC, which tend to focus on appearance (Oakes et al., 2017; Shafran et al., 2004), IAD-related BC might focus less on appearance and more on bodily signs of severe illnesses (Abramowitz & Moore, 2007). Therefore, IAD-related BC encompasses a detailed examination of disease-related symptoms such as noticeable moles or palpitations (Olatunji et al., 2011). Notably, performing BC may therefore vary according to disorder-specific characteristics (e.g., focus on appearance or health). Nevertheless, common to ED-, BDD-, and IAD-related BC is that in all three checking types, individuals might engage in one and the same behavior (e.g., looking in the mirror), albeit with different intentions, e.g., inspecting one’s shape in EDs, looking at flaws in BDD, or examining potentially conspicuous moles in IAD.

As a further commonality, cognitive behavioral models of all three disorders (i.e., EDs, BDD, and IAD) postulate a similar mechanism of BC, that is an initial short-lived relief from unpleasant emotions (cf. EDs: Fairburn et al., 2003; Williamson et al., 2004; BDD: Rosen et al., 1995; Wilhelm et al., 2014: IAD: Bleichhardt & Rief, 2014; Salkovskis & Warwick, 1986) but a subsequent longer-term maintenance of these emotions (Fairburn et al., 2003; Rachman et al., 1976; Rosen et al., 1995). It is assumed that individuals with EDs, BDD, or IAD, or individuals at risk of these disorders, mainly show BC in situations characterized by negative affect, presumably triggered by dysfunctional information processing (Warwick & Salkovskis, 1990; Wilhelm & Neziroglu, 2002; Williamson et al., 2004). By engaging in BC, the individual purportedly aims to reduce (or undo) this negative affect, and this reduction is negatively reinforced over time (Bleichhardt & Rief, 2014; Wilhelm et al., 2014; Williamson et al., 2004). Thus, in the short term, i.e., directly after a BC episode (cf. Opladen et al., 2022), the individual may feel reassured by BC, as performing the behavior may regulate overwhelming emotions (Veale, 2004).

In contrast to the theories (Bleichhardt & Rief, 2014; Wilhelm et al., 2014; Williamson et al., 2004), studies on the short-term emotion regulation function of BC have revealed an ambiguous picture. Regarding clinical populations of EDs, BDD, and IAD, only some studies found indications of the proposed short-term reduction of negative affect (Abramowitz & Moore, 2007; Hartmann et al., 2019; Kostopoulou et al., 2013), while others observed higher negative affect after BC (EDs: Suda et al., 2013; BDD: Veale et al., 2016; IAD: Doherty-Torstrick et al., 2016). Moreover, the existing studies examining ED-, BDD-, and IAD-related BC in nonclinical populations mostly did not confirm the model assumptions of a short-term reduction of negative affect. Indeed, with regard to ED-related BC, some studies even found a short-term increase – instead of the proposed decrease – in negative affect (Kraus et al., 2015; Pak et al., 2018; Suda et al., 2013; Tanck et al., 2019) and body dissatisfaction (Blechert et al., 2009; Smeets et al., 2011; Walker et al., 2021; Wilson et al., 2020). Similarly, regarding BDD-related BC, several studies have reported an increase in distress (Veale & Riley, 2001) and body dissatisfaction (Barnier & Collison, 2019; Veale et al., 2016; Windheim et al., 2011). In a study examining IAD-related BC, Hadjistavropoulos and Lawrence (2007) found that women with high health anxiety showed greater concern about their health following a stimulus to trigger illness anxiety compared to women with low health anxiety. The reasons for the discrepant results in studies analyzing the proposed short-term reduction of negative affect after BC remain unclear. The limited findings in support of the theory are complicated by the inherent association between engaging in the disorder-specific BC behavior on the one hand and the existence of the disorder itself on the other hand, thus casting uncertainty on the short-term effects of disorder-specific BC (e.g., appearance- or health-related) and in the absence of the psychopathology. Moreover, although theories propose that BC is predominantly performed in situations that are characterized by negative affect (e.g., Williamson et al., 2004), no research has experimentally induced negative affect before BC is performed.

The present study aimed to examine the theoretically proposed short-term consequences of BC (Bleichhardt & Rief, 2014; Wilhelm et al., 2014; Williamson et al., 2004) by comparing and disentangling disorder-specific BC features from the psychopathology of the various disorders themselves. Hence, we conducted a laboratory experiment with healthy females in which negative affect was induced in all participants before participants were randomized to one of three conditions and engaged in either ED-, BDD-, or IAD-related BC. To control for the effects on BC, we further applied a control checking condition (i.e., checking the characteristics of two vases). Following theoretical assumptions of a short-term reduction of negative affect by BC, we hypothesized that from the beginning to the end of each BC episode, all participants would show a short-term decrease in self-reported negative affect, with a stronger decrease in the BC groups (i.e., ED-related BC, BDD-related BC, IAD-related BC) compared to the control checking condition. Finally, from an exploratory perspective, we examined potential differences between ED-related, BDD-related, and IAD-related BC in terms of affective and cognitive reactions.

## Materials and Method

The present study is part of a larger project that is funded by the German Research Foundation (DFG), preregistered at the German Clinical Trials Register (ID: DRKS00025780) and at the Open Science Framework (ID: OSF: https://osf.io/rbvk4), and was approved by the first author’s institutional ethics committee (51/2019)

### Participants

Inclusion criteria were self-identifying as female, age between 18 and 65 years, the absence of a current mental disorder, and no past ED, BDD, or IAD, according to the Diagnostic Interview for Mental Disorders (Diagnostisches Interview bei psychischen Störungen, DIPS; Margraf et al., 2021). Exclusion criteria were underweight, defined as a body mass index (BMI) below 18.5 kg/m^2^ (WHO, 1995), current suicidal tendencies, self-harm behavior, and drug or substance abuse. We only included female participants as they have been found to show, on average, higher body dissatisfaction (Quittkat et al., 2019) and illness anxiety tendencies (Bleichhardt & Hiller, 2007) compared to a mixed-gender sample. During a telephone screening and the first appointment, *n* = 9 participants were screened out after the first interview as they showed an eating disorder in partial remission, current substance abuse, or had no further wish to continue the study. Therefore, a total of *N* = 102 women was eligible to participate in the study and were randomized to the three groups: *n* = 38 in the ED-related BC group, *n* = 32 in the BDD-related BC group, and *n =* 32 in the IAD-related BC group. As reimbursement, participants received course credits or a €10 gift voucher per hour of participation.

### Self-Report Measures

#### Screening Measures

##### Diagnostic Interview for Mental Disorders (DIPS)

During the first session, we applied the DIPS (*Diagnostisches Interview bei psychischen Sto¨rungen*; Margraf et al., 2021), a structured interview of approximately 60 – 120 minutes duration. It is adapted to the DSM-5 criteria, covering the most clinically significant mental disorders in adults. During the telephone interview, we screened for the absence of mental disorders, focusing in particular on the Mini-DIPS (Margraf & Cwik, 2017) questions relating to EDs, BDD, and IAD.

#### State Measures

##### Self-Assessment Manikin (SAM)

The SAM (Bradley & Lang, 1994) is a figure-rating self-report measure in which participants rate current emotional Valence on a nine-point Likert scale from 1 = *positive* to 5 = *negative*. Both dimensions showed excellent Cronbach’s α values in the present study (α = .85 for *Arousal;* α = .89 for *Valence*).

##### Positive and Negative Affect Schedule (PANAS)

In the present study, we used five specific items of the PANAS (Grühn et al., 2010), taken from the dimension Negative Affect, to assess the specific state emotions that occur during a BC episode, i.e., disgust, shame, anxiety, depressiveness, insecurity. These items were chosen by a team of experts, who, based on clinical experience, also added the emotion of helplessness as a potentially predominant emotion which has not yet been assessed in the context of BC. Items were rated on a five-point Likert scale from 1 = *not at all* to 5 = *extremely*. The subscale Negative Affect showed adequate internal consistency in a validation study (Thompson, 2007) and excellent internal consistency in the present study (α = .93).

##### Disorder-Specific Items

To assess the severity of psychopathology, we utilized a single item capturing the main preoccupation experienced by individuals with EDs, BDD, and IAD, with each group (i.e., ED-, BDD-, and IAD-related BC) completing only the item relevant for their assigned BC type. Specifically, for EDs, we assessed current dissatisfaction with one’s body size and shape (adapted from the Body Image States Scale; Cash et al., 2002); for BDD, we assessed the current conviction of having bodily flaws (adapted from the Fragebogen Körperdysmorpher Symptome, engl. Body Dysmorphic Symptoms Inventory; Buhlmann, Wilhelm, et al., 2010), and for IAD, we examined the current fear of having a serious illness (adapted from the modified Short Health Anxiety Inventory; Bailer et al., 2013). The items were rated on a scale from 1 = *I completely disagree* to 5 = *I completely agree*. The items showed excellent internal consistency in the present study (α = .97).

### Procedure

#### Diagnostic Assessment

Participants completed the study across two sessions encompassing a diagnostic assessment and a laboratory experiment. After providing informed consent, prospective participants underwent a structured telephone interview in which they were briefly screened for the predefined inclusion and exclusion criteria using the Mini-DIPS. If participants showed no signs of a mental disorder, they were invited to attend the first session, where they received information about the voluntary nature of participation and the anonymized data storage. Subsequently, they underwent a more in-depth interview to confirm the absence of a current mental disorder (i.e., using the DIPS). Following the interview, eligible participants were randomly assigned to one of the three BC groups (i.e., ED-, BDD-, or IAD-related BC) and were then provided with a general definition of BC and informed about the typical BC behavior/strategies exhibited by women with either EDs, BDD, or IAD.

#### Laboratory Experiment

The second session comprised the laboratory experiment, which lasted for approximately 2.5 h and was set up as a cross-over design. Participants performed the experiment by clicking through the instructions shown on the computer screen. All six blocks of the experiment lasted for 10 minutes, and participants completed the state questionnaire battery before and after each block. The first block consisted of a baseline of neutral mood induction (cf. Opladen et al., 2022). In the second block, all participants were exposed to a negative affect induction in a disorder-specific manner through an audio-guided imagery task simulating the experience of stepping out of the shower on a Saturday morning and feeling either too fat (ED-related), with a bodily flaw (BDD-related) or physically unwell (IAD-related). In the third block, participants either performed the group-specific BC task or the control checking condition, depending on the randomized assignment of sequence (i.e., BC first or control checking condition first). In the BC task, participants followed an audio guide tailored to the characteristics of the respective group: In the ED-related BC task, participants were guided through an imagery exercise in which they were asked to place selective attention on their upper thighs and stomach; in the BDD-related BC task, the objective was to attentively regard their nose and facial skin, and in the IAD-related BC task, participants were instructed to concentrate on the functioning of the cardiovascular system and their moles. In the control checking condition, all participants heard the same audio recording, in which they were asked to check the features (e.g., colors, haptics, shapes) of two vases. In the fourth block, a neutral mood was again induced by a second neutral landscape movie (cf. Opladen et al., 2022). Subsequently, the disorder-specific negative affect from the second block was again induced in the fifth block, and in the sixth block, participants were guided to engage in the other respective condition which they had not experienced during the third block (i.e., either the BC or control checking condition).

### Data Analysis

The statistical analyses were performed using the software IBM Statistical Package for the Social Sciences (version 29). To ensure a proper manipulation, we tested for differences between the two conditions regarding sequence (i.e., Sequence 1: BC condition first or Sequence 2: control checking condition first). For this purpose, we used separate 2 × 2 analyses of variance (ANOVA) with the dependent variable mood (i.e., PANAS, SAM_Valence_) and the between-subjects factor Sequence. In this analysis, we increased the α towards .10 to avoid alpha error accumulation (cf. Verhoeven et al., 2005). For all other analyses, the significance level was set at α = .05. As a second manipulation check, we tested whether the induction of negative mood was effective in all groups. For this purpose, we conducted a mixed 2 × 3 ANOVA with Greenhouse-Geisser correction with the within-subjects factor Time (i.e., pre-, post-mood induction) and the between-subjects factor Group (i.e., ED-related, BDD-related, and IAD-related checking). The successful induction of neutral mood was tested in a preliminary study (cf. Opladen et al., 2022). To test the hypothesized influence of BC on affect and pathology, we used mixed ANOVAs in a 2 × 2 × 3 design with the within-subjects factors Time and Condition (i.e., BC condition, control checking condition) and the between-subjects factor Group for the dependent variables. Post-hoc Bonferroni-corrected pairwise comparisons were employed. Effect sizes were classified into small ($\eta_{p}^{2}$ = 0.01), medium ($\eta_{p}^{2}$ = 0.09), and large ($\eta_{p}^{2}$ = 0.25; Lakens, 2013). The Shapiro-Wilk test was conducted to test the approximate normal distribution of the dependent variables, which was mostly met. As ANOVAs can be seen as generally robust to violations of normality (cf. Tabachnick & Fidell, 2007), we proceeded with the analysis even in cases in which variables were not normally distributed. The assumption of sphericity was assessed using Mauchly's test of sphericity, and Greenhouse‐Geisser correction was applied in the case of violation.

## Results

### Participant Characteristics and Manipulation Checks

Participants had a mean age in the early twenties (*M* = 23.01, *SD* = 4.07, Range = 18 – 51 years) and their mean BMI lay in the normal range (*M* = 22.60, *SD* = 3.43, Range = 18.07 – 37.66 kg/m^2^). First, as shown in Table 1, the results for all groups showed no effects of Sequence for any of the dependent variables. Moreover, further shown in Table 2, the second manipulation of an increase in negative affect from before to after mood induction was found in all groups as post-hoc pairwise comparisons revealed a negative difference between pre- and post-mood induction (Sequence 1: Δ_pre-post_ = -.19, *p* < .001; Sequence 2: -.24, *p <* .001), indicating an increase in negative affect in all groups in both sequences. This was also demonstrated by SAM_Valence_ as post-hoc pairwise comparisons revealed the increase in negative valence (Sequence 1: Δ_pre-post_ = -.733, *p* < .001; Sequence 2: Δ_pre-post_ = -.538, *p* < .001).

**Table 1:** ANOVAs for Manipulation Check for Sequence

Variable	Time Point	Body Checking Condition	Control Checking Condition
PANAS^a^	pre	F(5,67) = 1.69, *p* = .150, $\eta_{p}^{2}$ = .11	*F*(8,81) = 1.84, *p* = .182, $\eta_{p}^{2}$ = .15
	post	*F*(9,67) = 1.21, *p* = .305, $\eta_{p}^{2}$ = .14	*F*(3,81) = 1.07, *p* = .367, $\eta_{p}^{2}$ = .04
SAM_Valence_^b^	pre	*F*(3,90) = .12, *p* = .949, $\eta_{p}^{2}$ = .11	*F*(3,88) = 1.50, *p* = .224, $\eta_{p}^{2}$ = .05
	post	*F*(4,90) = 5.16, *p* = .724, $\eta_{p}^{2}$ = .02	*F*(3,88) = .85, *p* = .469, $\eta_{p}^{2}$ = .03
Pathology^c^	pre	*F*(4,87) = 1.46, *p* = .220, $\eta_{p}^{2}$ = .06	*F*(4,88) = .50, *p* = .736, $\eta_{p}^{2}$ = .02
	post	*F*(4,87) = 1.80, *p* = .137, $\eta_{p}^{2}$ = .08	*F*(4,88) = 1.19, *p* = .320, $\eta_{p}^{2}$ = .05

*Note.* Effect of Sequence: Body checking condition first vs. control checking condition first. Time Point refers to measurements before and after the respective condition.

^a^Specific items of the Positive and Negative Affect Schedule. Items were rated on a five-point Likert scale from 1 = *not at all* to 5 = *extremely*. ^b^Self-Assessment Manikin. Items were rated on a nine-point Likert scale from 1 = *aroused/positive* to 5 = *calm/negative*. ^c^Disorder-Specific Pathology. The items were rated on a scale from 1 = *I completely disagree* to 5 = *I completely agree*.

**Table 2:** ANOVAs for Manipulation Check for Negative Affect

Variable	Time Point	Sequence	Negative Mood Induction
PANAS^a^	pre – post	1	*F*(1, 99) = 26.75, *p* < .001, $\eta_{p}^{2}$ = .21
	pre – post	2	*F*(1, 99) = 36.80, *p* < .001, $\eta_{p}^{2}$ = .27
SAM_Valence_^b^	pre – post	1	*F*(1, 99) = 70.30, *p* < .001, $\eta_{p}^{2}$ = .42
	pre – post	2	*F*(1, 99) = 43.19, *p* < .001, $\eta_{p}^{2}$ = .30

*Note.* Effect of negative mood induction: Increase in negative affect before and after the mood induction. Time Point refers to measurements before and after the respective condition.

^a^Specific items of the Positive and Negative Affect Schedule. Items were rated on a five-point Likert scale from 1 = *not at all* to 5 = *extremely*. ^b^Self-Assessment Manikin. Items were rated on a nine-point Likert scale from 1 = *aroused/positive* to 5 = *calm/negative*.

### Effects of Body Checking

#### Negative Affect

Table 3 presents the *M* and *SD* for the dependent state variables for each group, condition, and time point, indicating generally low values for psychopathology in the sample. Numerical results of the ANOVAs are presented in Table 4.

**Table 3:** Means and Standard Deviations of All Dependent Variables for Each Group, Condition, and Time Point

*M* / *SD*	ED (*n =* 38)				BDD (*n =* 32)				IAD (*n =* 32)			
	BC		Non-BC		BC		Non-BC		BC		Non-BC	
	pre	post	pre	post	pre	post	pre	post	pre	post	pre	post
	PANAS^a^											
*M*	1.23	1.30	1.24	1.06	1.35	1.53	1.29	1.04	1.22	1.42	1.37	1.12
*SD*	0.46	0.59	0.53	0.20	0.55	0.60	0.38	0.11	0.41	0.50	0.58	0.39
	SAM_Valence_^b^											
*M*	2.42	2.37	2.34	2.16	2.78	2.75	2.59	2.22	2.56	2.66	2.63	2.25
*SD*	0.83	0.85	0.97	0.82	0.91	1.02	0.91	0.66	0.67	0.79	0.71	0.76
	Disorder-Specific Items											
*M*	2.03	2.34	2.00	1.58	1.97	2.34	1.81	1.41	1.28	1.50	1.34	1.22
*SD*	1.13	1.19	1.14	0.89	1.00	1.18	0.86	0.67	0.81	0.95	0.79	0.79

*Note.* pre / post refers to measurements before and after the respective condition. ED = Eating Disorder-Related Body Checking group; BDD = Body Dysmorphic Disorder-Related Body Checking group; IAD = Illness Anxiety Disorder-Related Body Checking group; BC = Body Checking Condition; Non-BC = Control Checking Condition.

**Table 4:** Analysis of Variance on the Effects of Body Checking

Outcome	PANAS^a^	SAM_Valence_^b^	Disorder-Specific Items
Main Effect			
Time	Λ = .97, *F*(1, 99) = 2.78, *p* = .089, $\eta_{p}^{2}$ = .03	Λ = .92, *F*(1, 99) = 9.07, *p* =.003, $\eta_{p}^{2}$ = .08	Λ = .01, *F*(1, 99) = .02, *p* =.885, $\eta_{p}^{2}$ = .00
Group	*F*(2, 99) = .54, *p* = 0.587, $\eta_{p}^{2}$ = .11	*F(*2, 99) = 1.32, *p* = .271, $\eta_{p}^{2}$ = .03	*F*(2, 99) = 5.66, *p* = .004, $\eta_{p}^{2}$ = .10
Condition	Λ = .78, F(1, 99) = 27.49 *p* < .001, $\eta_{p}^{2}$ = .22	Λ = .82, *F*(1, 99) = 22.67, *p* < .001, $\eta_{p}^{2}$ = .18	Λ = .72, *F*(1, 99) = 37.74, *p* < .001, $\eta_{p}^{2}$ = .28
Interaction			
Time × Group	Λ = .97, *F*(2, 99) = .20, *p* = .820, $\eta_{p}^{2}$ = .00	Λ = .96, *F*(2, 99) = .43, *p* =.782, $\eta_{p}^{2}$ = .01	Λ = .99, *F*(2, 99) = .35, *p* = .703, $\eta_{p}^{2}$ = .01
Condition × Time	Λ = .63, F(1, 99) = 57.83, *p* < .001, $\eta_{p}^{2}$ = .37	Λ = .91, *F*(1, 99) = 10.40, *p* = .002, $\eta_{p}^{2}$ = .96	Λ = .76, *F*(1, 99) = 30.63, *p* < .001, $\eta_{p}^{2}$ = .24
Group × Condition	Λ = .92, *F*(2, 99) = 4.18, *p* = .018, $\eta_{p}^{2}$ = .08	Λ = .96, *F*(2, 99) = 1.97, *p* =.145, $\eta_{p}^{2}$ = .04	Λ = .91, *F*(2, 99) = 4.81, *p* = .010, $\eta_{p}^{2}$ = .09
Time × Group × Condition	Λ = .97, *F*(2, 99) = 1.72 *p* = .185, $\eta_{p}^{2}$ = .03	Λ = .98, *F*(2, 99) = 1.06. *p* =.350, $\eta_{p}^{2}$ = .02	Λ = .97, *F*(2, 99) = 1.84, *p* = .231, $\eta_{p}^{2}$ = .03

*Note.* Time: Measurements before and after the respective condition; Group: Eating Disorder, Body Dysmorphic Disorder, and Illness Anxiety Disorder-Related Body Checking; Condition: Body Checking, Control Checking.

^a^Specific items of the Positive and Negative Affect Schedule. ^b^Self-Assessment Manikin.

First, regarding negative affect assessed using the PANAS, we found a significant main effect of Condition and significant interactions of Condition × Time and Group × Condition. Regarding Condition, post-hoc pairwise comparisons revealed a higher negative affect in the BC condition than in the control checking condition (Δ_BC-control checking_ = .155, *p* < .001). The interaction Group × Condition indicated that the ED-related and the BDD-related BC groups experienced a decrease in negative affect after the BC compared to the control checking condition (ED-related BC: Δ_BC-control checking_ = .116, *p* = .018, BDD-related BC: Δ_BC–control checking_ = .276, *p* < .001). Pairwise comparisons for the interaction Time × Condition indicated higher values after the BC condition (Δ_pre-post_ = -.150, *p* < .001) and lower values after the control condition (Δ_pre-post_ = .225 *p* < .001). For SAM_Valence_, the results revealed main effects of Time and Condition and a significant interaction of Time × Condition. Post-hoc, the main effect of Time indicated a higher value before than after each condition (Δ_pre–post_ = .154, *p* = .003). The main effect of Condition showed that negative valence was higher in the BC condition than in the control checking condition (Δ_BC–control checking_ = .225, *p* < .001). Regarding the interaction Time × Condition, negative valence was lower (i.e., more positive affect) after the control checking condition (Δ_pre–post_ = .311, *p* < .001).

#### Disorder-Specific Pathology

Regarding the disorder-specific ED, BDD, and IAD items, the analyses revealed main effects of Condition, Group, and an interaction of Time × Condition. Post-hoc comparisons for Condition revealed that participants undergoing the BC condition exhibited a higher level of disorder-specific pathology compared to those undergoing the control checking condition (Δ_BC–control checking_ = .350, *p* < .001). Regarding Group, pairwise comparisons revealed that the ED-related checking and BDD-related checking groups did not differ from each other, but that both groups differed from the IAD-related checking group, insofar as the latter group generally showed lower worry about their body in the current moment (Δ_IAD–ED_ = -.651, *p* = .006; Δ_IAD–BDD_ = -.547, *p* = .035). The interaction of Time × Condition revealed higher values after the checking in the BC condition and lower values in the control checking condition (BC condition: Δ_pre–post_ = -.303, *p* < .001; control checking Δ_pre–post_ = .317, *p* < .001).

## Discussion

The present laboratory experiment examined the short-term consequences of ED-related, BDD-related, and IAD-related BC with the goal of analyzing the assumption of negative reinforcement of BC, as proposed in etiological models of EDs (Williamson et al., 2004), BDD (Wilhelm et al., 2014), and IAD (Bleichhardt & Rief, 2014). To disentangle the BC behavior from the respective psychopathology, we compared the effects of disorder-specific types of BC in nonclinical women. In contrast to the model assumptions of a reduction of negative affect (Bleichhardt & Rief, 2014; Wilhelm et al., 2014; Williamson et al., 2004) from immediately before to after the BC condition, we found an increase in negative affect and disorder-specific pathology. Although the lack of short-term reduction of negative affect contradicts theoretical postulates, it is in line with the limited body of empirical research in nonclinical persons in the area of EDs, BDD, and IAD, which likewise did not observe a decrease in negative affect directly after a BC episode (e.g., Hadjistavropoulos & Lawrence, 2007; Kraus et al., 2015; Veale & Riley, 2001). These unexpected findings direct attention to the proposed root of checking behavior, which originates in the field of obsessive-compulsive disorder (OCD; Carr, 1974; Hodgson & Rachman, 1977). Our result of increased negative affect might be explained by potential differences in information processing between checking one’s own body, as is the case in ED-, BDD-, or IAD-related BC, and compulsive checking, as is typically performed by persons with OCD. During BC that is focused on negative evaluations of one’s own body (e.g., looking at generally unappreciated areas of the body), existing dysfunctional processing of body-related stimuli (Leonidou & Panayiotou, 2018; Neziroglu et al., 2008) and already developed body-related schemata (Williamson et al., 2004; Yan et al., 2019) may be activated. These schemata may in turn lead to an attentional bias towards disliked (Cordes et al., 2015) or potentially endangered (Witthöft et al., 2013) body parts, thus resulting in higher negative affect following ED-, BDD-, and IAD-related BC. In contrast, when checking objects, as performed by persons with OCD (e.g., checking that plugs on the stove are turned off to prevent fire; Veale & Roberts, 2014), none of these body-related schemata are activated. Thus, in line with considerations by Shafran et al. (2004), the results of the present study imply that the extension from compulsive checking to BC is not empirically applicable. Furthermore, we did not find significant differences between the diagnosis-specific types of ED-, BDD-, and IAD-related BC in the short-term activation of negative affect and psychopathology. Therefore, one can assume a potential transdiagnostic resemblance in the underlying mechanisms of BC across all three types of BC. However, worry about the body was lower in the IAD-related BC group compared to the ED- and BDD-related BC groups. This might be attributable to an initial vulnerability, suggesting that women may be more susceptible to psychopathology associated with appearance-related disorders (i.e., BN, BDD) compared to illness anxiety (i.e., IAD). This assumption is underscored by the higher prevalence rates observed for appearance-related disorders (cf. Buhlmann, Glaesmer, et al., 2010; Galmiche et al., 2019) compared to IAD (cf. Weck et al., 2014).

By contrast, we did find the postulated decrease in negative affect for the control checking condition (i.e., checking the characteristics of two vases). This finding is in line with a study by Walker et al. (2021), in which a 10-min non-BC condition (i.e., examining a text for errors) led to higher body satisfaction, self-esteem, and a marginally lower negative affect compared to a group that performed a 10-min critical BC condition. Thus, the potential cognitive distraction of checking *something* (i.e., vases in the present study) may reduce negative affect, while performing ED-, BDD-, and IAD-related BC, and the associated activation of body-related schemata, may not. The question of why BC is such a common behavior (Baptista et al., 2021) – if it is not negatively reinforced by negative affect – is thus still unanswered. In two studies examining participants with clinical EDs, BDD, and IAD (Hartmann et al., 2019) and nonclinical women (Opladen et al., 2022), the attainment of certainty, and not the reduction of negative affect, was noted as the most relevant function of BC. Thus, further studies might focus on the attainment of certainty as a potential reinforcing mechanism of BC. If our finding of an increase in negative affect is consistently replicated in clinical samples, we would support Guthoff et al. (2019) call for a revision of the prevailing theories (i.e., Bleichhardt & Rief, 2014; Wilhelm et al., 2014; Williamson et al., 2004), suggesting that the course of negative affect is not negatively reinforced, but that BC immediately leads to negative affective consequences.

### Limitations

Although the present study was the first to compare ED-, BDD-, and IAD-related BC in healthy women, allowing BC behavior to be disentangled from its associated psychopathology, certain limitations need to be considered when interpreting the results. First, we only included healthy females. Future studies should therefore examine a more heterogenous sample, although previous research has shown similar results, for example, in samples with healthy men regarding BDD-related BC (Cordes et al., 2017; Walker et al., 2012) and IAD-related BC (Hadjistavropoulos et al., 1998). Furthermore, as our participants did not have a mental disorder, our results cannot yet be transferred to clinical samples of individuals with EDs, BDD, and IAD. Second, we cannot be certain that participants deployed their attention towards the regions of their body that they were supposed to look at. However, according to administered manipulation check items, most participants generally examined their body closely and did not use strategies to distract themselves from the task. Third, for reasons of comparability and in reference to Windheim et al. (2011), all participants checked their body for 10 min. However, the length of a BC episode in real life might differ idiosyncratically. Further, we only examined BC with a focus on negative evaluations of one's own body performed in a standardized manner (i.e., each group inspected two specific body areas related to BN-, BDD-, and IAD-associated BC). However, different types and variations in the execution of BC may occur, which were not addressed in our experiment, thus limiting the generalizability of our findings to all forms of body checking. To enhance ecological validity, future studies might take this into account and adjust the time interval and type for which participants are instructed to perform BC.

### Implications

In sum, across all diagnosis-specific BC types, a short-term relief of negative affect after BC, as proposed in the etiological models (Bleichhardt & Rief, 2014; Wilhelm et al., 2014; Williamson et al., 2004), was not confirmed by the present findings. Instead, negative affect increased after ED-, BDD-, and IAD-related BC. The results of our study suggest that the theories may need to be adapted, as assumptions regarding checking behavior in OCD (i.e., Carr, 1974; Hodgson & Rachman, 1977) might not be transdiagnostically valid for BC characteristics in EDs, BDD, and IAD. The increase in negative affect after BC instead shows that BC has negative effects not only in the longer term (Opladen et al., 2022), but also in the short term, further underlining the dysfunctionality of BC.

## Supplementary Materials

The Supplementary Materials contain the preregistration for the study (see Hofschröer et al., 2023S).



HofschröerV.
VivellM.-B.
SchulzI.
HirschfeldG.
HartmannA. S.
VocksS.
 (2023S). Does body checking regulate emotions over time? Psychophysiological and cognitive-affective effects of appearance- and health-related body checking
[Preregistration]. PsychOpen. https://osf.io/rbvk4


## Data Availability

The data and materials that support the findings of this study are available on request from the corresponding author, Vanessa Hofschröer, without undue reservation.

## Reporting Guidelines

Our research adheres to the Journal Article Reporting Standards (JARS) for quantitative research in psychology.

## Preregistration

The present study is part of a project that is funded by the German Research Foundation (DFG) that is preregistered at the German Clinical Trials Register (ID: DRKS00025780). The study is registered at the Open Science Framework (https://osf.io/rbvk4).

## Abbreviations

BC = Body Checking; ED = Eating Disorder; BDD = Body Dysmorphic Disorder; IAD = Illness Anxiety Disorder; DIPS = Diagnostic Interview for Mental Disorders; SAM = Self-Assessment Manikin; PANAS = Positive and Negative Affect Schedule.
